# Pharmacological targeting of ROS reaction network in myeloid leukemia cells monitored by ultra-weak photon emission

**DOI:** 10.18632/oncotarget.23175

**Published:** 2017-12-11

**Authors:** Rosilene Cristina Rossetto Burgos, Rawi Ramautar, Eduard P.A. Van Wijk, Thomas Hankemeier, Jan Van Der Greef, Alireza Mashaghi

**Affiliations:** ^1^ Leiden Academic Centre for Drug Research, Faculty of Mathematics and Natural Sciences, Leiden University, 2300 RA Leiden, The Netherlands; ^2^ Sino-Dutch Centre for Preventive and Personalized Medicine/Centre for Photonics of Living Systems, Leiden University, 2300 RA Leiden, The Netherlands

**Keywords:** reactive oxygen species, ultra-weak photon emission, NADPH oxidase, myeloperoxidase, pharmacological inhibitors

## Abstract

Acute myeloid leukemia (AML) is a blood cancer that is caused by a disorder of the process that normally generates neutrophils. Function and dysfunction of neutrophils are key to physiologic defense against pathogens as well as pathologies including autoimmunity and cancer. A major mechanism through which neutrophils contribute to health and disease is oxidative burst, which involves rapid release of reactive oxygen species (ROS) generated by a chemical reaction network catalyzed by enzymes including NADPH oxidase and myeloperoxidase (MPO). Due to the involvement of neutrophil-derived reactive oxygen species in many diseases and importance of NADPH oxidase and MPO-mediated reactions in progression and treatment of myeloid leukemia, monitoring this process and modulating it by pharmacological interventions is of great interest. In this work, we have evaluated the potential of a label-free method using ultra-weak photon emission (UPE) to monitor ROS production in neutrophil-like HL60 myeloid leukemia cells. Suppression of ROS was achieved by several drug candidates that target different parts of the reaction pathway. Our results show that UPE can report on ROS production as well as suppression by pharmacological inhibitors. We find that UPE is primarily generated by MPO catalyzed reaction and thus will be affected when an upstream reaction is pharmacologically modulated.

## INTRODUCTION

Innate immune cells are key to health and many diseases. Neutrophil granulocytes (also called neutrophils), the most abundant innate immune cells, are at the forefront to fight against infections, regulate the adaptive immune system, and contribute to tissue damage when activated in excess [1–4]. During phagocytosis, neutrophils react to microbes, virus, and bacteria releasing several types of oxidants to kill the invading pathogens. The respiratory burst is the first mechanism of defense during phagocytosis and requires oxygen (O_2_) consumption to produce and release reactive oxygen species (ROS) [5]. The rapid release of superoxide anion radicals (O_2_^·-^) and hydrogen peroxide (H_2_O_2_), which are the primary source of the oxidants, is followed by rapid conversion into other oxidant species (OH·, HOCl, etc) [5]. These processes are catalyzed mainly by two enzymes, NADPH oxidase and myeloperoxidase (MPO), the latter being a signature protein of neutrophils. Physiologically, ROS production is beneficial at right doses; however, the overproduction of ROS (usually called as oxidative stress) has been related to several disorders such as Alzheimer's disease [6], Parkinson's disease [7], cancer [8, 9], cardiovascular diseases [10] and chronic diseases such as diabetes [11], and rheumatoid arthritis [12].

Acute myeloid leukemia (AML) is a blood cancer that is caused by a disorder of the process that normally generates neutrophils [13]. AML is most commonly seen in adults and is associated with high morbidity and mortality [14]. Mutations in receptor tyrosine kinases (RTKs) and its downstream effectors are believed to underlie this cancerous process [15]. MPO is a lineage marker for acute myeloid leukemia and can serve as a prognostic factor. On the other hand, NADPH oxidase-derived reactive oxygen species serves as an immune evasion strategy by which AML cells kill the healthy immune cells. In brief, NADPH oxidase and MPO-mediated reactions are important in progression and treatment of AML.

Given the involvement of ROS in many diseases, drug therapies which target specific sites of ROS production are getting attention [16–18]. Various antioxidants and specific inhibitors of NADPH oxidase have been developed in recent years as a promising target for treating several types of cardiovascular diseases such as atherosclerosis [18, 19]. MPO inhibitors have also been considered as new potential drugs [20, 21]. MPO is the downstream pathway of NADPH oxidase, acting only at inflammation sites [22]. The overproduction of oxidants species by MPO has been reported to cause tissue damage and others complications in several diseases [23]. Modulation of ROS response could also be beneficial in AML therapy as well as in tissue destruction caused by excessive recruitment and activation of neutrophils.

In this work, we propose a label-free method using ultra-weak photon emission (UPE) to monitor pharmacological inhibition of ROS machinery in AML (HL-60) cells. UPE is endogenous light emitted by human tissues and is believed to be related to ROS generation [24, 25]. This weak light is emitted in the ultraviolet/visible range (100 – 800nm) possibly reaching the near-IR spectrum (801 – 1300nm) and originates from radiative (non-thermal) electronic transitions of excited electron states during reactions with biomolecules [24, 25]. Due to the close relation of UPE and ROS generation, UPE can be used as a dynamic monitoring tool for oxidative metabolism [26, 27]. In this work, we used three classes of drugs, namely anti-oxidants, specific NADPH oxidase inhibitors and an MPO inhibitor and monitored their response by UPE analysis. This analysis demonstrates whether or not UPE can report on the activity of these drugs and reveals reactions that primarily generate the emitted light.

## RESULTS

### Monitoring ROS by UPE measurement

**Figure 1:**
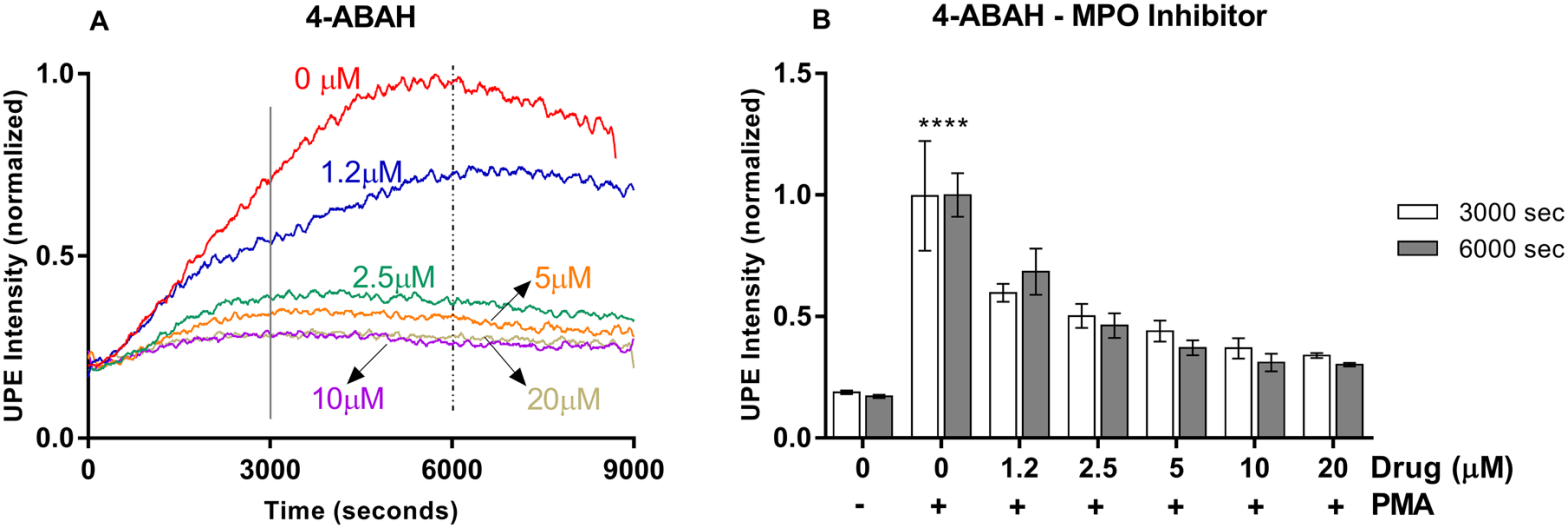
A representative UPE profile of HL-60 cells in resting state and upon triggering ROS response stimulated by PMA UPE profile was recorded for 9000 seconds at 37°C in the dark. The lines represent the smoothed UPE intensity followed by normalization by the highest UPE intensity.

We first demonstrate that AML cells generate UPE upon triggering ROS response [26]. We find that AML cells generate a weak UPE signal in resting state and this signal is amplified when the cells are treated with PMA [28]. Figure 1 shows a representative time trace of UPE during PMA stimulation of AML cells (see also Supplementary Figure 1). PMA is known to induce respiratory bursts in AML and neutrophils, thus we attribute the recorded UPE signal to ROS response [26].

**Figure 2:**
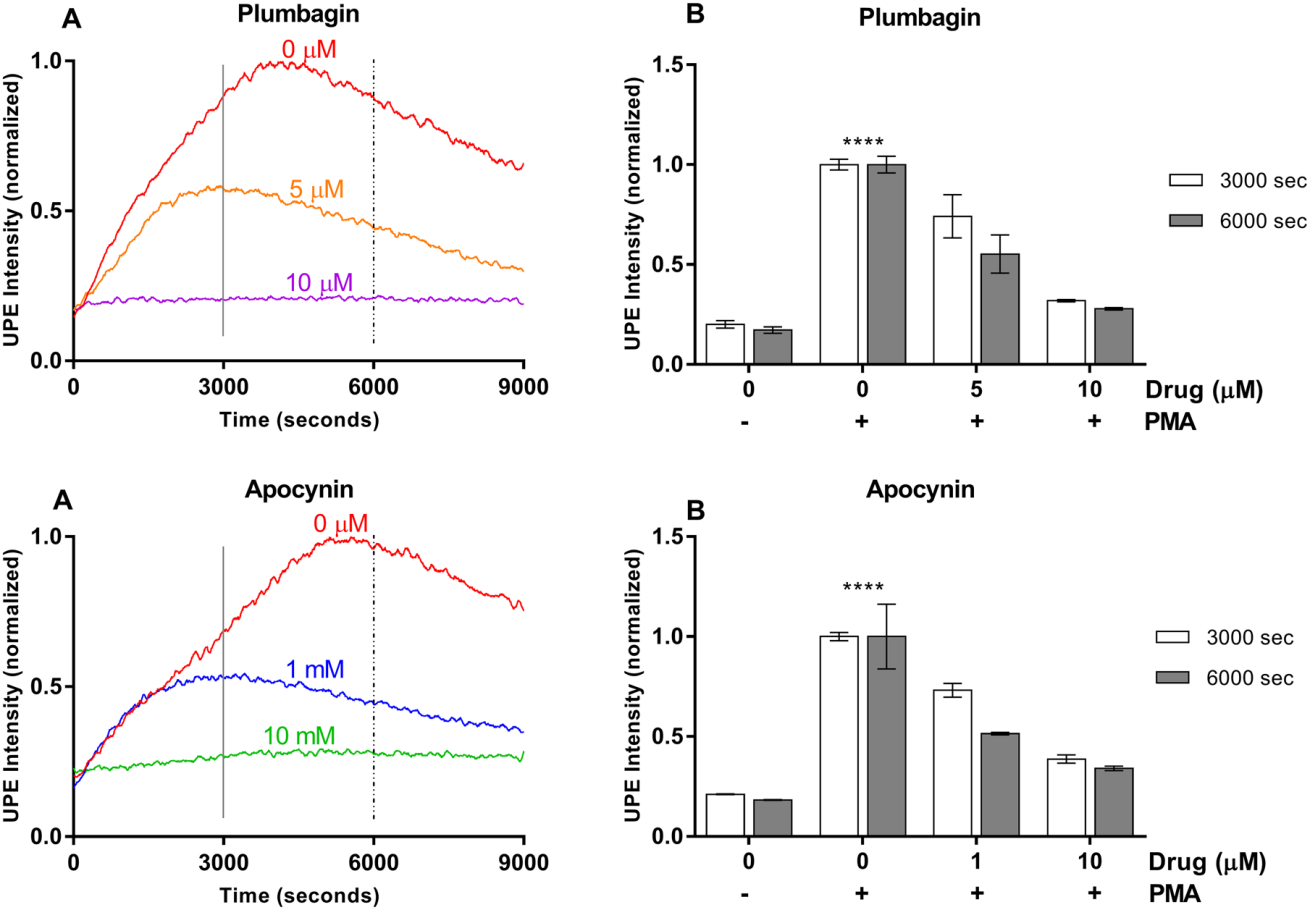
Plumbagin and Apocynin effects on UPE profile in HL-60 cells **(A)** Dynamic UPE profileshowing the suppression of UPE intensity with the administration of scavengers Plumbagin and Apocynin in two different concentrations (n=1). **(B)** Analysis of the interval (3000 – 3600 seconds and 6000 – 6600 seconds) as indicated in (A) by the vertical lines. Statistical significance was determined by two-way ANOVA with errors bars represented as standard deviation (SD) and n≥3. ^****^p<0.0001.

To better demonstrate the link between the recorded signal and the ROS process, we quenched ROS response by two scavengers namely plumbagin and apocynin. We observed that UPE signal gets suppressed significantly by administration of these drugs (Figure 2). These lines of evidence clearly show that UPE analysis can detect ROS response and antioxidant activities in AML cells. Our experiments above show that UPE can inform about oxidative metabolism, but it does not provide any molecular or pathway information. Plumbagin and apocynin scavenge ROS and also non-specifically inhibit the enzymatic reaction pathway that leads to ROS generation [29–36]. To provide mechanistic insights, in the following we target the reaction network using specific inhibitors of the key elements involved in ROS generation.

### Targeting NADPH oxidase

**Figure 3:**
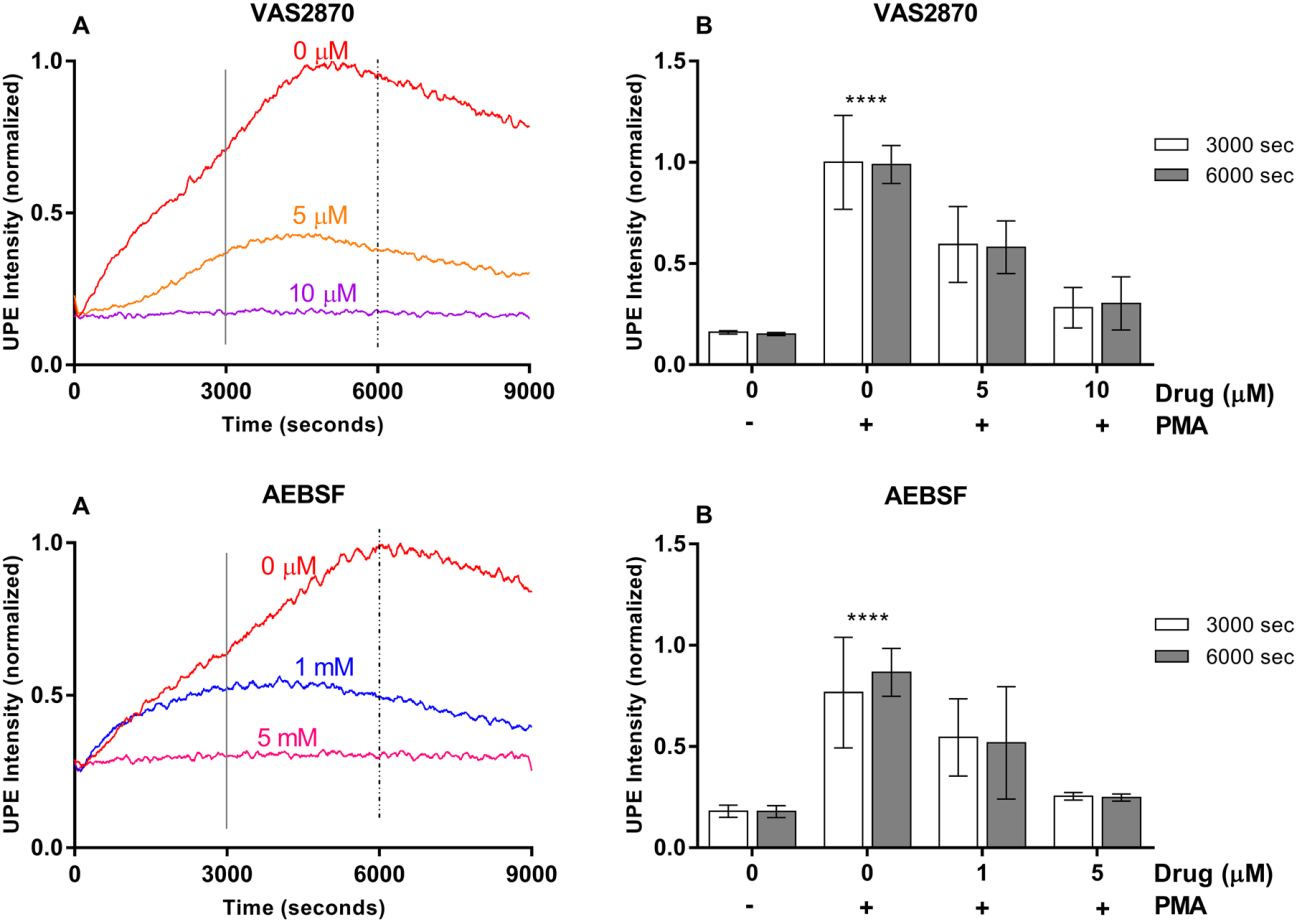
VAS2870 and AEBSF effects on UPE profile in HL-60 cells **(A)** Dynamic UPE profileshowing the suppression of UPE intensity with the administration of NADPH oxidase inhibitor VAS2870 and AEBSF in two different concentrations (n=1). **(B)** Analysis of the interval as indicated in (A) by the vertical lines. Statistical significance was determined by two-way ANOVA with errors bars represented as standard deviation (SD) and n≥3.

To gain better mechanistic and molecular insights, we investigated if UPE reports on ROS pathway downstream or upstream (or both) to NADPH oxidase. To address this question, we inhibited NADPH oxidase specifically (Figure 3) and checked whether the UPE signal is affected or not. Two specific drugs were tested and the results indicate that downstream processes to NADPH oxidase contribute to the recorded UPE signal. By increasing the concentration of the drugs we could fully block the UPE signal, indicating that nearly all of the UPE signal is due to NADPH oxidase-mediated reaction and/or downstream processes with no detectable contributions from upstream or parallel reactions. This finding raises the question whether UPE signal is due to neutrophil specific MPO-mediated reaction or it is caused by non-specific reactions, i.e. NADPH oxidase- and/or superoxide dismutase (SOD)-mediated reactions (Supplementary Figure 2).

### Targeting myeloperoxidase

**Figure 4:**
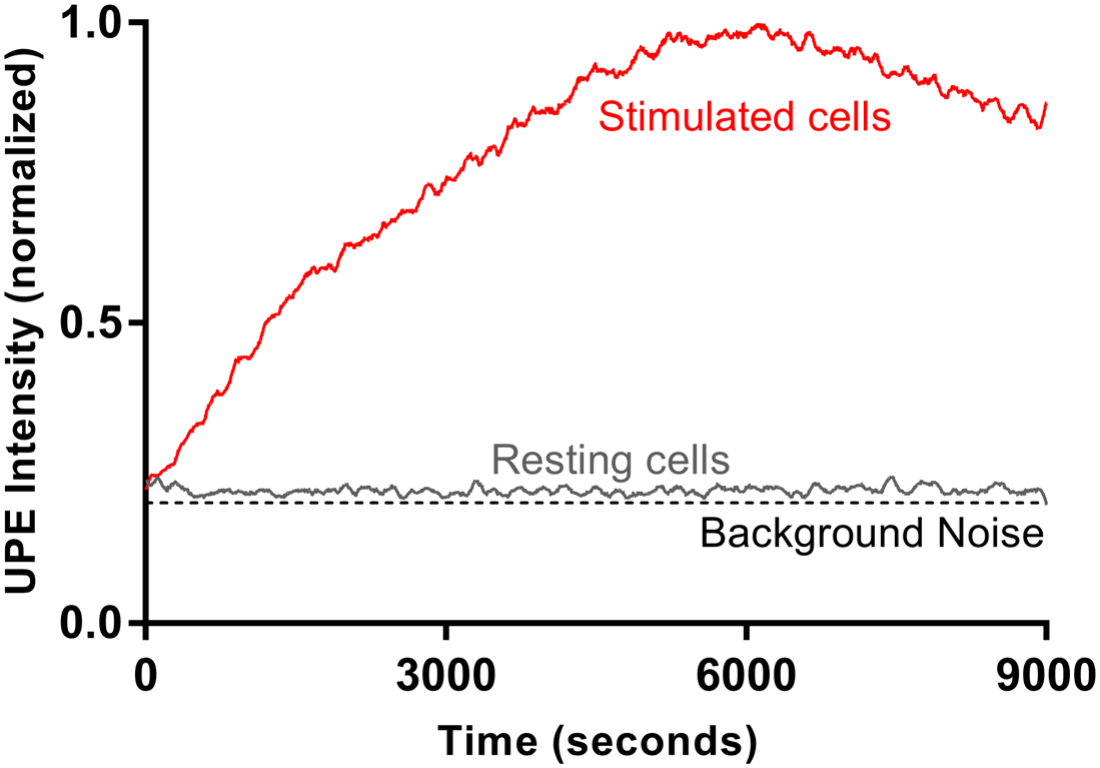
MPO inhibitor tested in the HL-60 cell model system measured by UPE **(A)** Dynamic UPE profileshowing the suppression of UPE intensity with the administration of 4-ABAH in five different concentrations (n=1). **(B)** Analysis of the interval as indicated in (A) by the vertical lines. Statistical significance was determined by two-way ANOVA with errors bars represented as standard deviation (SD) and n≥3. ^****^p<0.0001.

Next, we aimed to resolve the contribution of neutrophil-specific MPO-mediated reactions to the UPE signal, a reaction which is downstream to the NADPH oxidase- and SOD-mediated processes in the ROS reaction network. For this aim, we tested a specific MPO inhibitor and measured the UPE signal. If the signal is caused by a process that depends on NADPH oxidase or SOD but not on MPO, we expect no effect by MPO inhibition. Figure 4 presents the results obtained for the MPO inhibitor 4-ABAH tested. Intriguingly, MPO inhibition clearly suppressed UPE signal. By increasing the concentration of MPO inhibitor we could nearly reach a full suppression. The results clearly indicate that UPE reports on ROS processes that are downstream to MPO catalysis and as such UPE can specifically report on MPO activity.

## DISCUSSION

UPE has been considered a potential tool to monitor dynamic oxidative metabolism, but its utilization for medical diagnostics and pharmacology still requires more insights into the mechanism and the biochemical pathways that drive its generation. For this aim, we modulated ROS pathways pharmacologically and monitored UPE in time. We focused our study on neutrophil-like cell HL-60 [37–40] because ROS generation by neutrophils is critically important in disease processes including cancer, infection, and tissue destruction in excessive immune responses. We tested several NADPH oxidase inhibitors with a wide range of specificity (antioxidants, NOX, etc.) and also the downstream pathway specific for neutrophils (see Supplementary Figure 2) using an irreversible MPO inhibitor.

Our results show that UPE was able to monitor ROS production and suppression in all potential drug candidates tested independently of the specificity of the inhibitor. In addition, UPE response was dose-dependent for all drugs tested and in agreement with the IC_50_ found in the literature. Importantly, we have also checked cell viability during the drug treatment period of 9000 seconds recorded by UPE being the cells with a great viability during the time recorded (see Supplementary Figure 3). Our analysis indicates that the UPE signal can be fully suppressed when one of the few parallel pathways that form the ROS reaction network is blocked. ROS reaction network involves not only NADPH oxidase-MPO pathway but also the xanthine oxidase and mitochondrial pathways [41–43]. The fact that blocking NADPH oxidase-MPO pathway leads to full suppression of the UPE signal (see Figures 2, 3, 4) suggests that contributions from other reactions are negligible. Thus, specific reporters need to be designed and used to monitor other pathways in ROS reaction network.

Our pharmacological manipulation of ROS reaction pathway in AML cells and monitoring the outcome by UPE revealed that UPE can report on ROS generation and suppression. Our data indicate that MPO-mediated reaction is mainly responsible for the UPE signal. This is a unique capability for UPE because it provides a very simple, low-cost, label-free method for providing dynamic information on MPO-mediated ROS response. The application of this technology will not be limited to AML, where MPO-mediated ROS response can be used as a prognostic measure, but also in other cancers where tumor-associated neutrophils suppress T cell immunity via generation of ROS response [4, 44].

## MATERIALS AND METHODS

### Cell culture, differentiation, and induction of the respiratory burst in HL-60 cells

Acute promyelocytic leukemia cell line – HL-60 (catalogue number CCL-240; lot number 62690063; ATCC, Manassas, VA) was cultured in Iscove's Modified Dulbecco's Medium – IMDM without phenol red (Gibco-Life Technologies, Grand Island, NY), supplemented with 10% (v/v) of fetal calf serum (FCS) and 1% (v/v) penicillin/streptomycin (Sigma-Aldrich, St. Louis, MO). Cell seed and maintenance were kept between the exponential growths (2×10^5^ - 1.0×10^6^ cells per ml) in a CO_2_ incubator at 37 °C. The cell count and viability (>85%) was determined using the trypan blue exclusion method with an automated cell counter (Bio-Rad Laboratories, Hercules, CA). For the differentiation into *neutrophils-like* cells, we have used the standard protocol as described previously [27, 26]. In brief, when the cells were split and adjusted for cell density, 1 μM all-trans retinoic acid (ATRA; 98% grade, catalog number R250, Sigma-Aldrich) was added to the cells in order to induce differentiation via the granulocytic pathway. The cells were incubated for up to 7 days, and UPE experiments were performed on day 7. Cells were stimulated with 54 nM of phorbol 12-myristate 13-acetate – PMA (98% grade, Sigma-Aldrich, St. Louis, MO) in the presence or absence of inhibitors: 4-Aminobenzoic acid hydrazide – 4-ABAH (Cayman Chemicals, Ann Arbor, MI); 4-(2-Aminoethyl)benzene sulfonyl fluoride hydrochloride – AEBSF; 1,3-Benzoxazol-2-yl-3-benzyl-3H-[1, 2, 3]triazolo[4,5-d]pyrimidin-7-yl sulfide – VAS2870; 5-hydroxy-2-methyl-1,4-naphthoquinone – Plumbagin; 4-Hydroxy-3-methoxyacetophenone – Apocynin (Sigma-Aldrich, St. Louis, MO). Measurements were performed between cell passage numbers P07 - P28. As a standard protocol in immunology, ROS generation in neutrophil or neutrophil-like cells is typically assessed following stimulation by PMA and comparing it to ROS response by the cells in their resting state [28].

### Ultra-weak photon emission (UPE) measurement

A 2-inch photomultiplier tube – PMT (series 9558B with S20 photocathode) purchased from ET Enterprises (Sweetwater, TX) was used for the UPE measurements. The detector was cooled to -25°C in order to reduce the noise. The photon emission intensity was recorded over the time (counts/sec). A Peltier element was used inside the dark chamber to maintain the sample at 37°C and the PMT was set in a vertical position at the top of the dark chamber (see Supplementary Figure 4). UPE was measured in HL-60 cells after PMA (54nM) induction. For each UPE measurement, a small aliquot of the cell suspension (6 ml at a cell density of 1×10^6^ cells/ml) was used to record the UPE profile. First, the background was measured for 1000 seconds before PMA induction and subsequently, cells were stimulated with PMA in the presence or absence of inhibitors for 9000 seconds.

### Myeloperoxidase and NADPH oxidase inhibitors

We have used HL-60 cells differentiated into *neutrophil-like* cells and induced a respiratory burst by applying phorbol 12-myristate 13-acetate (PMA). The respiratory burst was recorded for 9000 seconds and the potential of several NADPH inhibitors (VAS2870, Plumbagin, AEBSF, and Apocynin) and the irreversible myeloperoxidase inhibitor (4-ABAH) were evaluated. Apocynin, AEBSF, VAS2870, Plumbagin, and 4-ABAH were added at the indicated concentrations prior PMA induction. Only AEBSF was added 15 minutes before PMA stimulation.

### Data analysis

UPE data were processed and plotted using the software GraphPad Prism 7.0. The UPE data were smoothed using the function xy analysis (smooth - 2^nd^ order of smoothing with 100 neighbors data points). Thus, the smoothed data were normalized by the highest UPE intensity (See the appended Supplementary Table 1). The smoothed curve is presented as dynamic data. Next, we have analyzed specific regions of the dynamic data (3000-3600 seconds and 6000-6600 seconds) to generate statistics averaging the smoothed data. Normalization was done by the average value of the UPE intensity induced only by PMA. Two-way ANOVA followed by Tukey multiple comparison tests with GraphPad Prism 7 was used to identify significant differences. Differences with a *p*-value<0.05 were considered significant.

## SUPPLEMENTARY MATERIALS FIGURES AND TABLES




