# Supplementary material for: Pharmacological targeting of ROS reaction network in myeloid leukemia cells monitored by ultra-weak photon emission
Source: Oncotarget. 2017 Dec 11;9(2):2028–34. doi: 10.18632/oncotarget.23175 (PMC5788618; doi:10.18632/oncotarget.23175)
Supplement: Supplementary file 1 [file oncotarget-09-2028-s001.pdf]

# Pharmacological targeting of ROS reaction network in myeloid leukemia cells monitored by ultra-weak photon emission

## SUPPLEMENTARY MATERIALS

## REFERENCES

1. Hideg E, Inaba H (1991) Biophoton emission (ultraweak photoemission) from dark adapted spinach chloroplasts. Photochemistry and photobiology 53:137-142.
2. SŁAWIŃSKA D, SŁAWIŃSKI J (1987) Ultraweak photon emission in model reactions of the *in vitro* formation of eumelanins and pheomelanins. Pigment Cell & Melanoma Research 1:171-175.

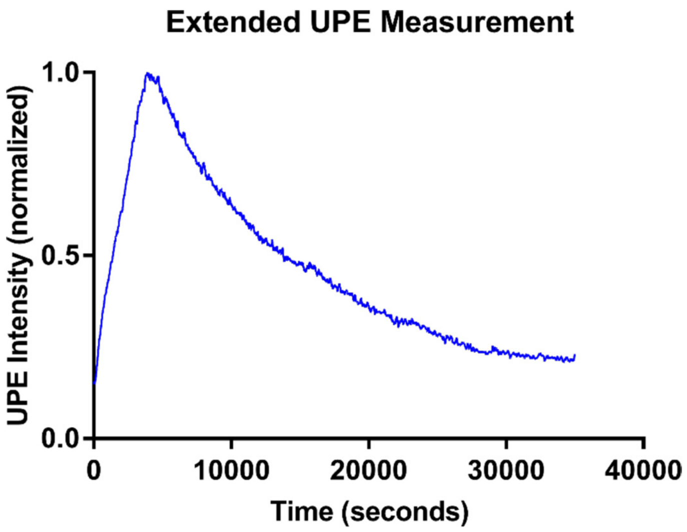


**Supplementary Figure 1: Long-term UPE measurement.** UPE profile of HL-60 cells upon triggering ROS response stimulated by PMA. UPE profile was recorded for 35000 seconds (about 9.7 hours) at 37°C in the dark. The lines represent the smoothed UPE intensity followed by normalization by the highest UPE intensity.

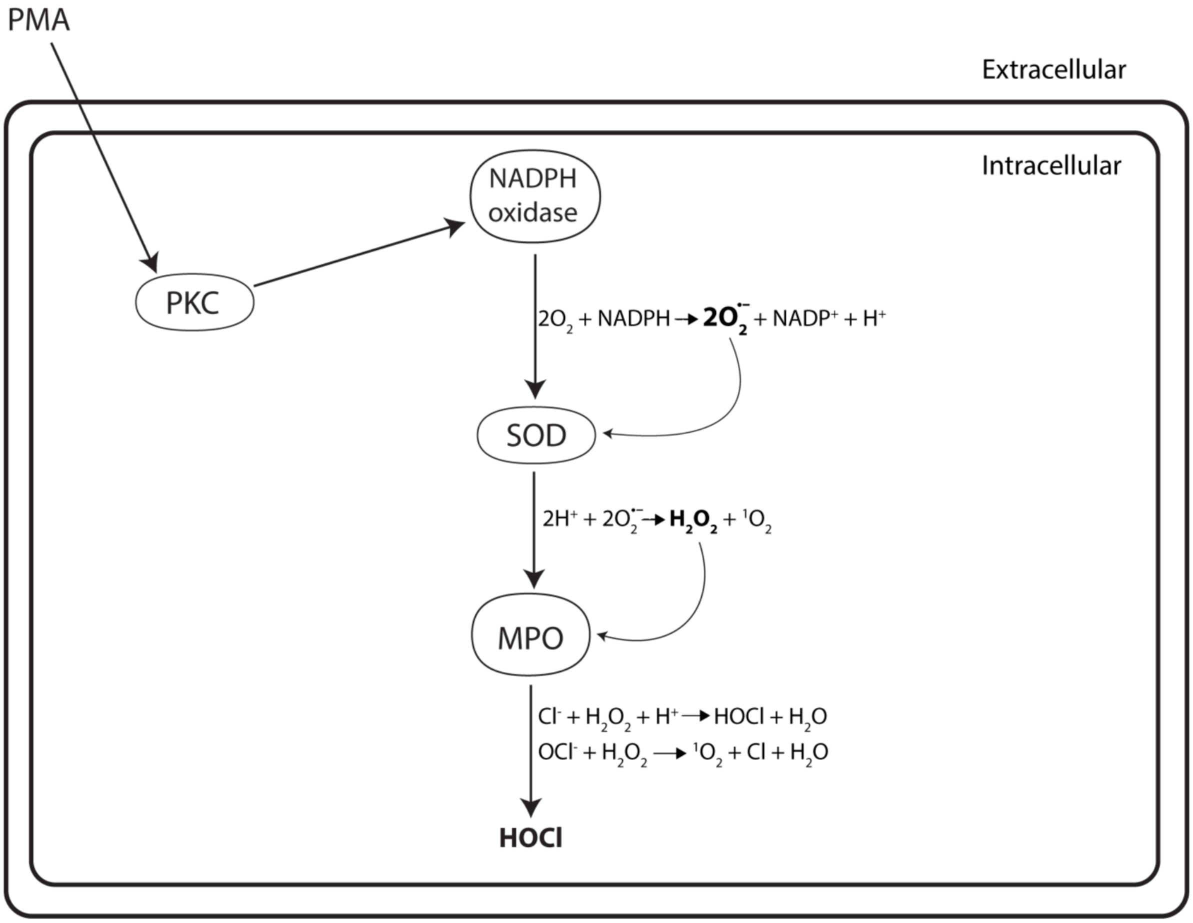


**Supplementary Figure 2: Downstream ROS pathway NADPH – MPO enzymatic system.** In our HL- 60 cells model, PMA activates protein kinase C (PKC) and NADPH oxidase. Activated NADPH oxidase transfers electrons from NADPH coupling to molecular oxygen in order to produce superoxide anion as a primary ROS source which is quickly dismutated to H2O2 by superoxide dismutase (SOD). Peroxide is the substrate for myeloperoxidase (MPO) which catalyzes the oxidant production (e.g. HOCl) to kill pathogens at the inflammation sites. In this study, we targeted PKC with PMA, NADPH oxidase with a few specific and non-specific inhibitors, and MPO with a specific inhibitor. Our findings and conclusions are in agreement with previous studied where SOD is targeted [1,2].

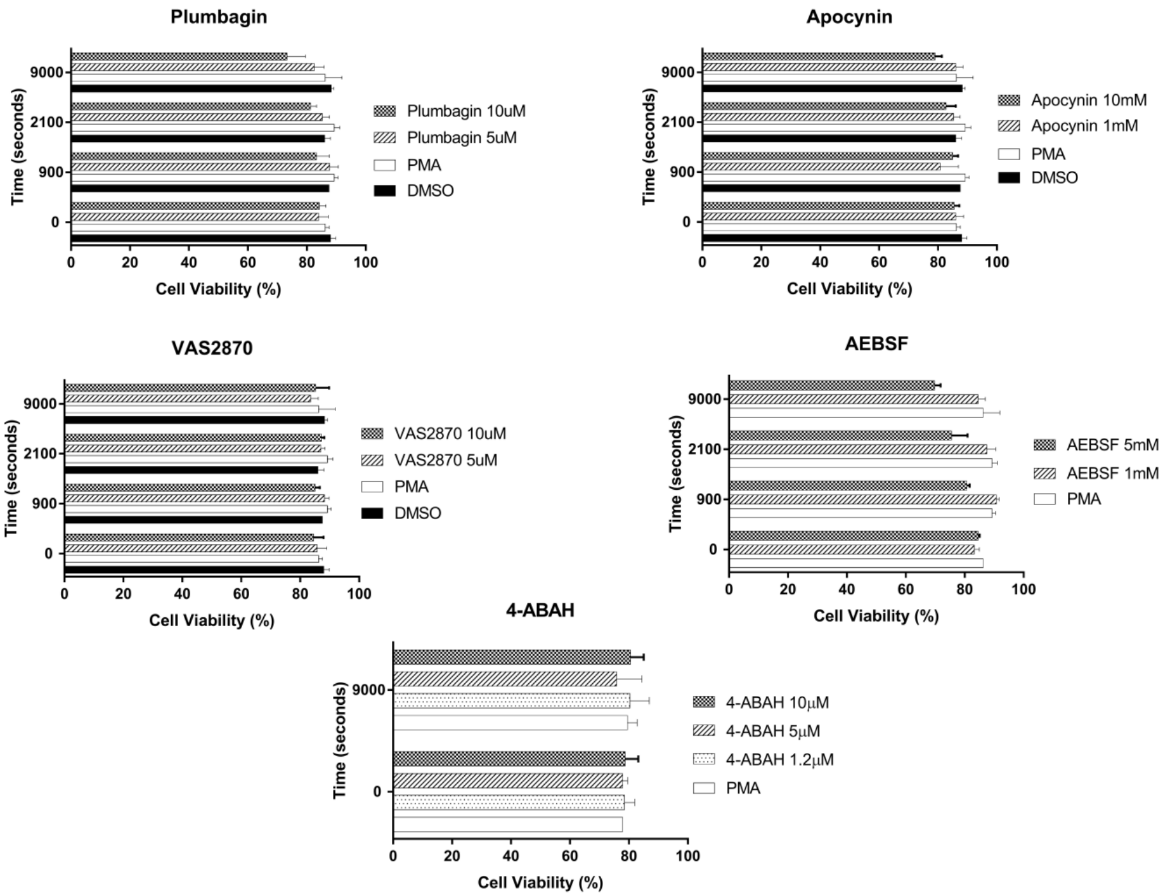


**Supplementary Figure 3: Cell Viability during drug administration in HL-60 cells.** The viability was checked during 900, 2100 and 9000 seconds for antioxidants and NADPH oxidase inhibitors (Plumbagin, Apocynin, VAS2870 and AEBSF) and at 9000 seconds for MPO inhibitor (4-ABAH). In some drug administration, we also tested the vehicle (DMSO) when necessary.

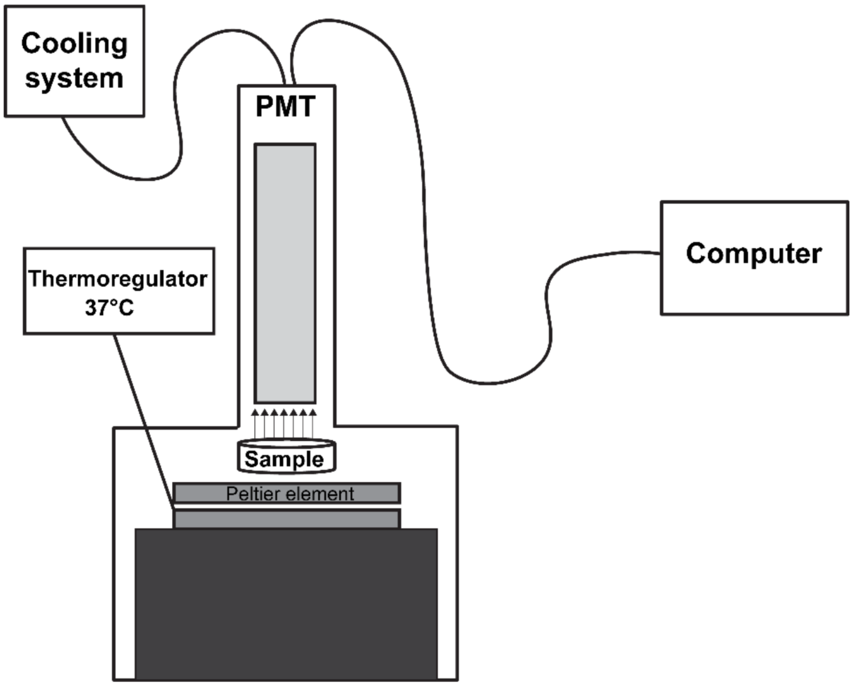


**Supplementary Figure 4: Instrument set up for UPE measurements.** A PMT was used for the UPE measurements was set in a vertical position at the top of the dark chamber. The detector was cooled to -25°C in order to reduce the noise. A Peltier element was used inside the dark chamber to maintain the sample at 37°C.

**Supplementary Table 1: Raw Data**

See Supplementary File 1
